# 15-Deoxy-Delta-12,14-prostaglandin J2 modulates pro-labour and pro-inflammatory responses in human myocytes, vaginal and amnion epithelial cells

**DOI:** 10.3389/fendo.2022.983924

**Published:** 2022-09-21

**Authors:** Zahirrah BM. Rasheed, Yun S. Lee, Sung H. Kim, Tg Teoh, David A. MacIntyre, Phillip R. Bennett, Lynne Sykes

**Affiliations:** ^1^ Imperial College Parturition Research Group, Department of Metabolism, Digestion and Reproduction, Imperial College London, London, United Kingdom; ^2^ Universiti Kebangsaan Malaysia (UKM) Medical Molecular Biology Institute (UMBI), Universiti Kebangsaan Malaysia, Kuala Lumpur, Malaysia; ^3^ March of Dimes European Preterm Birth Prematurity Research Centre, Imperial College London, London, United Kingdom; ^4^ The Parasol Foundation Centre for Women’s Health and Cancer Research, St Mary’s Hospital, Imperial College Healthcare National Health Service (NHS) Trust, London, United Kingdom

**Keywords:** nuclear factor - kappa B (NF - κB), activator protein (AP)-1, inflammation, cytokines, prostaglandins, preterm labour (PTL), 15dPGJ2

## Abstract

**Background:**

Prematurity is the leading cause of childhood death under the age of five. The aetiology of preterm birth is multifactorial; however, inflammation and infection are the most common causal factors, supporting a potential role for immunomodulation as a therapeutic strategy. 15-Deoxy-Delta-12,14-prostaglandin J2 (15dPGJ2) is an anti-inflammatory prostaglandin and has been shown to delay lipopolysaccharide (LPS) induced preterm labour in mice and improve pup survival. This study explores the immunomodulatory effect of 15dPGJ2 on the transcription factors NF-κB and AP-1, pro-inflammatory cytokines, and contraction associated proteins in human cultured myocytes, vaginal epithelial cell line (VECs) and primary amnion epithelial cells (AECs).

**Methods:**

Cells were pre-incubated with 32µM of 15dPGJ2 and stimulated with 1ng/mL of IL-1β as an *in vitro* model of inflammation. Western immunoblotting was used to detect phosphorylated p-65 and phosphorylated c-Jun as markers of NF-κB and AP-1 activation, respectively. mRNA expression of the pro-inflammatory cytokines IL-6, IL-8, and TNF-α was examined, and protein expression of COX-2 and PGE2 were detected by western immunoblotting and ELISA respectively. Myometrial contractility was examined *ex-vivo* using a myograph.

**Results:**

15dPGJ2 inhibited IL-1β-induced activation of NF-κB and AP-1, and expression of IL-6, IL-8, TNF-α, COX-2 and PGE2 in myocytes, with no effect on myometrial contractility or cell viability. Despite inhibiting IL-1β-induced activation of NF-κB, expression of IL-6, TNF-α, and COX-2, 15dPGJ2 led to activation of AP-1, increased production of PGE2 and increased cell death in VECs and AECs.

**Conclusion:**

We conclude that 15dPGJ2 has differential effects on inflammatory modulation depending on cell type and is therefore unlikely to be a useful therapeutic agent for the prevention of preterm birth.

## 1 Introduction

Preterm birth (PTB) occurs in 5-18% of pregnancies worldwide and is the leading cause of mortality in children under the age of five (1, 2). There is a global drive to reduce the rates of preterm birth and its related morbidity and mortality. The United Nations Sustainable Development Goal 3 has set a target to reduce neonatal and under-five mortality to 12 and 25 per 1000 births respectively in all countries by 2030 (3). One of the challenges for reducing PTB rates is its multifactorial aetiology, and the unrealistic expectation that a single preventative strategy, for example tocolytics, could be effective regardless of aetiology. Infection and inflammation are the most common causal factors for spontaneous PTB, especially at earlier gestations, where morbidity and mortality are at their highest (4–6). Despite this knowledge, there have not been any new developments in therapies since the last century.

Term labour is an inflammatory process, with evidence of leukocyte invasion and pro-inflammatory cytokine production in the myometrium, cervix, and fetal membranes (7–11). In addition, pro-labour mediators COX-2, prostaglandins, matrix metalloproteinases contribute to fetal membrane activation, uterine contractility and cervical remodelling that is required for fetal membrane rupture, uterine contractions, and cervical dilation. The transcription factors NF-κB and AP-1 have been shown to regulate the expression of pro-inflammatory cytokines and pro-labour mediators such as COX-2, prostaglandins, and the oxytocin receptor (12–17). We and others have demonstrated *in vitro and ex vivo* activation of the transcription factors NF-κB and AP-1 by Toll like receptor (TLR) agonists (18–21), which leads to further augmentation of a pro-inflammatory response *via* a feed forward loop. Early activation of these transcription factors by microbes and/or cytokines is likely to trigger preterm cervical dilatation, fetal membrane rupture, myometrial contractility, and ultimately preterm birth, and therefore serve as potential therapeutic targets. The sources of infection can be *via* haematogenous spread, disseminated locally *via* the placenta, or most commonly ascending to the intrauterine cavity and fetal membranes from the vagina (22). It is plausible that therapeutics could be developed to target local sites, either the myometrium (systemic administration, or utilising nanoparticle technology) or the cervical-vaginal interface and overlying fetal membranes using a vaginal pessary.

15-Deoxy-Delta-12,14-prostaglandin J2 (15dPGJ2) is an endogenous prostaglandin of the J2 series (23, 24) and is a ligand for the peroxisome proliferator-activated receptor (PPAR)-γ, and components of the NF-κB, and AP-1 signalling pathways (25–27). Several studies have demonstrated differential expression of the PPAR receptors (28, 29), NF-κB (14, 30) and AP-1 (31–33) in gestational tissue, depending on gestational age and the presence or absence of labour. We have previously demonstrated that the anti-inflammatory prostaglandin 15-Deoxy-Delta-12,14-prostaglandin J2 (15dPGJ2) inhibits NF-κB at multiple levels in the NF-κB pathway in human myocytes and amnion epithelial cells (AECs) *in vitro* (26). Additionally, we have shown in a mouse model that 15dPGJ2 inhibits LPS-induced activation of NF-κB, delays LPS-induced preterm labour, and improves pup survival (20). In contrast, the effects of 15dPGJ2 on AP-1 activation *in vitro* has been shown to be variable, depending on cell type (27, 34–36). Given that AP-1 is a key mediator of inflammation-induced preterm labour in the mouse (16, 17, 37), it is important to establish the effect of 15dPGJ2 on AP-1 activation on human gestational tissue. We hypothesised that 15dPGJ2 would have immunosuppressive effects on human gestational tissue, and thus serve as a novel potential therapeutic agent for PTB prevention. We report on the effects of 15dPGJ2 on the transcription factors NF-κB and AP-1, pro-inflammatory cytokines and pro-contractile mediators in cultured human myocytes, vaginal and amnion epithelial cells.

## 2 Materials and methods

### 2.1 Ethics statement

Myometrial biopsies and placentas were collected from women undergoing elective caesarean section between 38- and 41-weeks gestational age at Queen Charlotte’s and Chelsea Hospital, Imperial College Healthcare NHS Trust, London. Consent was taken in accordance ethical committee approval from Riverside Research Ethics Committee (Ref 3358) and Hammersmith, Queen Charlotte’s & Chelsea Hospitals Research Ethics Committee (Ref 2002/628) and in accordance with Imperial College NHS Healthcare Trust Research and Development. Written consent was obtained from all subjects. Women undergoing pre-labour elective caesarean section from healthy pregnancies were selected. This was to avoid any confounding effects of labour and gestational age, such as variations in the expression of prostaglandin pathway and immune mediator genes (38), or pathologies, leading to variations in tissue responses.

### 2.2 Cell culture and treatments

#### 2.2.1 Myometrial cells

Myometrial biopsies were taken from the upper margin of lower segment incisions during elective caesarean sections. The tissue was washed with phosphate buffered saline (PBS) to rinse off excess blood and then dissected mechanically using two sterile blades to form a fine paste-like texture. Cells were isolated by incubating the tissue for 45 min at 37°C with a sterile enzyme mix of 10 mg of Collagenase 1A, 10 mg of Collagenase XI, and 200 mg of bovine serum albumin (BSA) in 30 mls of 1:1 of Dulbecco’s Modified Eagle’s Medium (DMEM) and F-12 HAM. DMEM containing 10% fetal calf serum (FCS) was added to inactivate the enzymes before the suspension was filtered through a cell strainer and centrifuged at 3000 rpm for 5 min. The pellet was resuspended in DMEM (10% FCS) containing 2 mM/L L-glutamine, 100 U/ml penicillin, and 100 µg/ml of streptomycin and cultured to confluence. Cells up to passage five were used for experiments. A minimum of 3 biological replicates were used per experiment.

#### 2.2.2 Vaginal epithelial cells

The vaginal epithelial cell line VK2/E6E7 was purchased from ATCC (ATCC^®^ CRL2616™) and stored in liquid nitrogen until use. In preparation for experiments, cells were thawed for 1 min at 37°C in a water bath and transferred into pre-warmed DMEM containing 10% FCS. The suspension was centrifuged at 125 x g for 5 min and cells were resuspended in keratinocytes serum free medium (KSFM), bovine pituitary extract (BPE), epidermal growth factor (EGF) and calcium chloride (CaCl_2_) at 37°C with 5% CO_2_ and grown to confluence. Confluent cells were trypsinised and plated for experiments. Only passage numbers 3-6 were used for experiments. Experiments were repeated a minimum of three times.

#### 2.2.3 Amnion epithelial cells

AECs were processed as described previously (19). Placentas were collected within an hour of delivery and processed immediately. The amnion tissue was separated from the choriodecidua, and the excess blood was removed by rinsing the tissue in PBS. The tissue was cut into strips of approximately 5cm length and then incubated in 0.5 mM EDTA-PBS for 15 minutes at room temperature. The strips were then washed with PBS and incubated in 60 mls of pre-warmed Dispase (Gibco) at 2 g/L in PBS for 50 min at 37°C. The strips were carefully removed from the Dispase and transferred into DMEM containing 10% FCS, 2 Mm/L L-glutamine, 100 U/ml Penicillin and 100 µg/ml Streptomycin, and shaken vigorously to isolate the epithelial cells. The floating strips were removed, and the medium was centrifuged at 2000 rpm for 10 min. The cell pellet was re-suspended in DMEM and filtered through a 20 µm filter. The collected cells were re-suspended to a required volume in DMEM containing 10% FCS, 2 Mm/L L-glutamine, 100 U/ml Penicillin and 100 µg/ml Streptomycin and plated for culture at 37°C with 5% CO_2_ until confluent. Confluence was reached by days 4 or 5.

### 2.3 Cell treatment

A concentration of 32 µm of 15dPGJ2, (Cayman Chemical, Ann Arbor, MI), was selected on the basis of previous data demonstrating inhibitory effect on p-p65 in peripheral blood mononuclear cells (PBMCs) (39),myocytes (26), and amniocytes (40). Although no published data exist for the optimal concentration to be used in VECs, we selected the same concentration for consistency, especially with the long-term view of needing to use single concentration/dosing regime in clinical practice. A vehicle control of DMSO was used for non-treated samples. Myocytes, VECs, and AECs were treated with 32 µM of 15dPGJ2 for 2 hrs before stimulation with IL-1β (1 ng/mL), (R&D Systems, Abingdon, UK). IL-1β (1 ng/mL) stimulation was for 15 mins when determining activation of NF-κB and AP-1, 4 hrs when determining expression of COX-2, and 24 hrs for PGE2 concentrations. A time course of 4, 8, 12 and 24 hrs was carried out when examining IL-6, IL-8, and TNF-α mRNA expression. Experiments were terminated by removing cell culture supernatant and storing cells at -80°C until further use. Between 3-6 biological replicates were performed for each experiment.

### 2.4 Protein extraction, Sodium Dodecyl sulfate polyacrylamide gel electrophoresis (SDS- PAGE) and Western immunoblotting

The adherent cells were lysed with whole cell lysis buffer (Cell Signalling) with 5 µl/mL of protease inhibitor cocktail (Sigma), 5 µl/mL pf phosphatase inhibitor cocktail (Sigma), 1 mM of PMSF and collected using a cell scraper. Cells were incubated on ice for 5 min and centrifuged at 14,000 g for 10 min at 4°C. Supernatant was collected and the protein was quantified using Bio-Rad quantification assay measuring absorbance at 655 nm (Bio‐Rad, Hercules, CA). Protein was resolved using SDS-PAGE and transferred onto PVDF membranes (GE Healthcare, Chalfont St Giles, UK) at a constant voltage of 300 mA. The membranes were then blocked in 5% (weight/volume) milk in Tris‐buffered saline supplemented with 0·1% Tween 20 for 1 hr at room temperature before incubation with primary and secondary antibodies as shown in **Table 1**. The chemiluminescence detection was performed using Clarity™ Western ECL Substrate (Bio-Rad) and imaged using chemiluminescent imager (ImageQuant) and the densitometry was performed with ImageQuantTL (version 8.1).

**Table 1:** Western immunoblotting antibodies.

	Primary antibody	Catalogue number	Secondary antibody	Catalogue number
**COX-2**	1:2000 o/n 4°C	Sc-1745 (Santa Cruz)	Anti-goat 1:2000 1hr RT	SC-2020 (Santa Cruz)
**P-p65 (Ser536)**	1:1000 o/n t 4°C	3031	Anti-rabbit 1:2000 1hr RT	7074
**P-c-Jun (Ser73)**	1:2000 o/n 4°C	9164	Anti-rabbit 1:2000 1hr RT	7074
**P-JNK1/JNK2 (Thr183/Tyr182)**	1:1000 o/n 4°C	9252	Anti-rabbit 1:2000 1hr RT	7074
**P-p38 (Thr180/Tyr182)**	1:1000 o/n 4°C	9211	Anti-rabbit 1:2000 1hr RT	7074
**P-ERK (Thr202/Tyr204)**	1:1000 o/n 4°C	9101	Anti-rabbit 1:2000 1hr RT	7074
**β-actin**	1:160,000 30 mins RT	AB6276 (ABCAM)	Anti-mouse1:8000 15mins RT	SC-2005

COX-2: cyclooxygenase-2; P-p65, phosphorylated p65; P-c-Jun, phosphorylated c-Jun; P-JNK, phosphorylated JNK; P-p38, phosphorylated p38; P-ERK, phosphorylated ERK; o/n, overnight; RT, room temperature.

### 2.5 Cytokine mRNA quantification by quantitative RT-qPCR

Total RNA was isolated from the adherent cells using Trizol^©^ (Invitrogen Life Technologies, Grand Island, NY) according to the manufacturer’s instructions. 2 µg of RNA was added to the mixture of 1 µl of DNAse 10x buffer and 1 µl of DNAse with DEPC treated water made to total volume of 10 µl. The solution was incubated for 15 min at room temperature before adding 1 µl of stop solution. The mixture was heated to 70°C for 10 min. 8 µl of the DNAse treated samples were then added to the remaining 1 μl 10 mM dNTP mix and 1 μl of Oligo DT (0.5 μg/ml), mixed and incubated at 70°C for 10 min. Later, 2 μl 10x M-MLV RT buffer, 1 μl M-MLV RT, 0.5 μl RNase Inhibitor, and 6.5 μl DEPC treated water were then added and the solution was incubated at room temperature for 15 min, 37°C for 50 min, and 90°C for 10 min. The resulting cDNA was diluted 1:5 for the specific amplification of genes of interest, and the fold change of target genes were analysed taking into account the reference gene β actin, **Table 2**. cDNA synthesis and RT-qPCR reagents were purchased from Sigma-Aldrich (Gillingham, UK) unless otherwise stated. Cycling conditions were; Polymerase activation at 94°C for 3 minutes, denaturing at 94°C for 45 s, annealing at 60°C for 30 s, extension at 72°C for 1 min 30 s, holding at 72°C for 10 minutes.

**Table 2:** Primers for gene expression.

	Forward 5’	Anti-sense 5’	Size (bp)
**COX-2**	TGTGCAACACTTGAGTGGCT	ACTTTCTGTACTGCGGGTGG	77
**cPLA2-α**	TGCATTCTGCACGTGATGTG	CACCATGGCTCGGAAACC	68
**IL-6**	CCTTCCAAAGATGGCTGAAA	AGCTCTGGCTTGTTCCTCAC	153
**IL-8**	GCCTTCCTGATTTCTGCAGC	CGCAGTGTGGTCCACTCTCA	151
**TNF-α**	TCCTTCAGACACCCTCAACC	CAGGGATCAAAGCTGTAGGC	208
**β-actin**	AGGCATCCTCACCCTGAAGTA	CACACGCAGCTCATTGTAGA	105

### 2.6 PGE2 quantification

Supernatant was collected after pre-treatment with either vehicle control or 15dPGJ2 and 24 hrs of IL-1β stimulation for quantification of PGE2 production by ELISA according to the manufacturer’s guidance (KGE004B, R&D Systems, Minneapolis, MN). The supernatant was diluted 1:3 with the assay diluent and plated alongside the calibration curve standards (range of 39-2,500 pg/mL). The optical density of each well was read at 450 nm, 540 nm and 570 nm. To calculate PGE2 concentrations, the average readings from the absorbance values of 540 and 570 nm was subtracted from the reading taken from 450 nm.

### 2.7 Myometrial contractility

Myometrial tissue biopsies were dissected at 4°C into 8 strips of approximately 5 mm x 10 mm and mounted onto the thermostatically-controlled isolated organ baths (DMT Myograph 800MS) and stretched to 4 g of tension. The tissue was allowed to equilibrate for 90 min in 4 mls of oxygenated (95% O2 and 5% CO2) modified Kreb’s solution (D-Glucose 2.0 g/L, Magnesium sulphate (anhydrous) 0.141 g/L, Potassium phosphate monobasic 0.16 g/L, Potassium chloride 0.35 g/L, Sodium chloride 6.9 g/L, Calcium chloride dihydrate 0.373 g/L, Sodium bicarbonate 2.1 g/L) at 37°C, pH 7.4. Strips were treated with a cumulative dose response from 1 µM to 100 µM of 15dPGJ2 after spontaneous contractions were established. At the end of the experiment 10^-7^ M of oxytocin was added to demonstrate myometrial strip contractility as a marker of viability. The total area under the curve, average area under the curve, average peak amplitude, and the rate of contractions were analysed by comparing to the vehicle control using the Powerlab software V5.5.6 (ADI Instruments) using the peak parameters extension.

### 2.8 Cell integrity and viability

Cell morphology was assessed every four hrs during incubations with 15dPGJ2 or vehicle control using standard light microscopy. The release of the LDH into myocyte culture medium using the LDH assay kit (Cayman Chemicals) was performed as per the manufacturer’s instructions with Triton-X (1%) as the positive control. Since the morphological appearance of VECs and AECs changed following incubation with 15dPGJ2, cell viability was determined using the MTT assay. Briefly, 50,000 cells were cultured in a 96 wells plate and MTT reagent was added into each well after treatment and incubated for 4 hrs at 37°C with 5% CO_2_. The crystal dissolving solution was then added into each well for 12 hrs at 37°C with 5% CO_2_ before reading the plate at 570 nm.

### 2.9 Statistical analysis

Statistical analyses were performed using Kruskal Wallis, one-way analysis of variance (ANOVA), or two-way ANOVA with Dunn’s or Bonferroni’s multiple comparison test depending on the distribution of data and the number of treated groups or tests on respective experiments. All analyses, including tests for normality were carried out using GraphPad v5 (GraphPad Software v5, San Diego, CA). A value of p<0.05 was considered to indicate statistical significance.

## 3 Results

### 3.1 Effects of 15dPG2 on myocytes

#### 3.1.1 15dPGJ2 inhibits IL-1β-induced transcription factor NF-κB and AP-1 in myocytes

**Figure 1:**
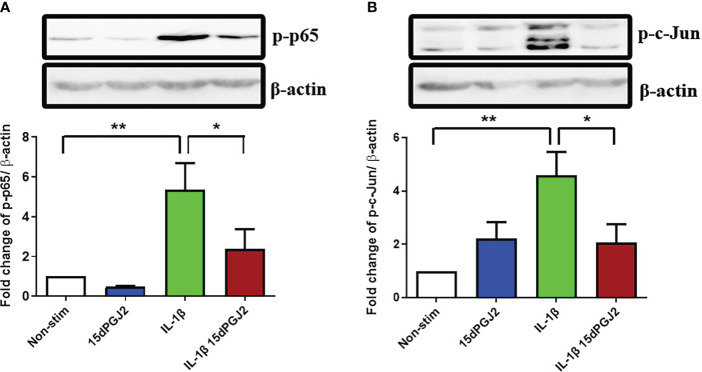
15dPGJ2 inhibits IL-1β-induced NF-κB and AP-1 activation in myometrial cells. Myometrial cells were pre-incubated for 2 hours with 32µM 15dPGJ2 or vehicle control before stimulation with 1ng/ml of IL-1β for 15 minutes. Whole cell lysates were prepared and assessed by Western immunoblotting for serine 536-phosphorylated p65 (Ser536-p-p65) as a marker of NF-κB activation, and serine 73-phosphorylated c-Jun (Ser73-p-c-Jun) as a marker of AP-1 activation. Representative immunoblots are shown for p-p65 **(A)** and p-c-Jun **(B)** with β-actin as a loading control. Densitometry analysis of the immunoblots showed a significant increase in p-p65 and p-c-Jun with IL-1β stimulation after 15 minutes (*p*<0.01). Pre-incubation with 15dPGJ2 significantly inhibited IL-1β-induced p-p65 and p-c-Jun (*p*<0.05) activation. n=5-6. *p<0.05, **p<0.01. Data are presented as mean ± SEM.

To study the effect of 15dPGJ2 on the activation of the transcription factors NF-κB and AP-1 in myocytes, cells were pre incubated with 15dPGJ2 or vehicle control for 2 hrs, and then stimulated with 1 ng/ml IL-1β for 15 min. We demonstrated that IL-1β stimulation leads to an increase in p-p65 (*p<*0.01), reflecting activation of NF-κB, and that 15dPGJ2 inhibits this effect (*p<*0.05), (**Figure 1A**). Similarly, IL-1β stimulation leads to an increase in p-c-Jun (p<0.01), reflecting activation of AP-1, and 15dPGJ2 inhibits this effect (*p*<0.05, **Figure 1B**).

#### 3.1.2 15dPGJ2 inhibits IL-1β-induced inflammatory mediators IL-6, IL-8, and TNF-α in myometrial cells

**Figure 2:**
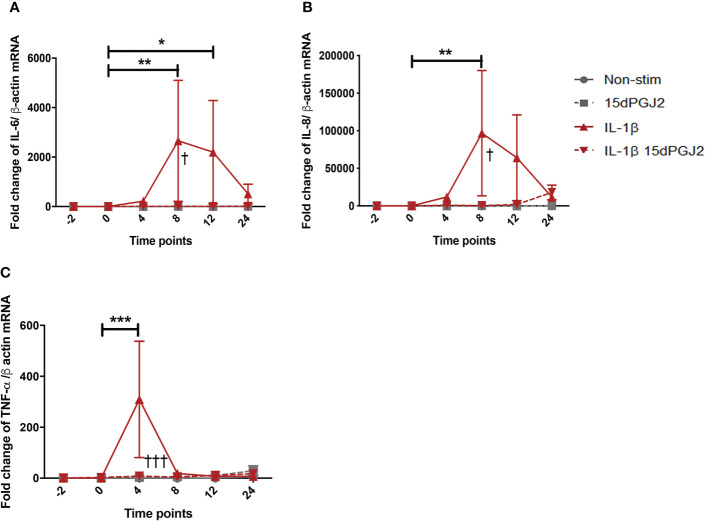
15dPGJ2 inhibits IL-1β-induced IL-6, IL-8, and TNF-α cytokines mRNA production in myometrial cells. Myometrial cells were pre-incubated for 2 hours with 32µM 15dPGJ2 or vehicle prior to 1ng/ml of IL-1β stimulation. Cells were harvested at -2, 0, 4, 8, 12 and 24 hours post IL-1β stimulation. Quantitative RT-qPCR was used to determine the effect of 15dPGJ2 on IL-6, IL-8, and TNF-α mRNA. Fold change took into account the reference gene β actin. A significant increase in IL-6 was observed with IL-1β at 8 and 12 hours (p < 0.01, p < 0.05), and inhibition of this effect was seen when cells were pre-incubated with 15dPGJ2 at 8 hours (p < 0.05) **(A)**. A significant increase in IL-8 was seen at 8 hours (p < 0.01), and inhibition of this effect was seen when cells were pre-incubated with 15dPGJ2 at 8 hours (p < 0.05) **(B)**. TNF-α mRNA was upregulated at 4 hours with IL-1β stimulation (p < 0.001), the effect of which was inhibited on pre-incubation with 15dPGJ2 (p < 0.001) **(C)**. n = 3. The effect of IL-1β was compared to -2 non-stimulated (vehicle) time point. *p < 0.05, **p < 0.01, ***p < 0.001. The effect of 15dPGJ2 was determined by comparing the IL-1β plus 15dPGJ2 treatment to IL-1β alone ^†^p < 0.05, ^†††^p < 0.001. Data are presented as mean ± SEM.

IL-1β stimulation significantly increased IL-6 mRNA at 8 (*p*<0.01) and 12 (*p*<0.05) hrs, the effect of which was significantly inhibited by 15dPGJ2 (**Figure 2A**). Similarly, IL-1β induced IL-8 expression at 8 hrs (*p*<0.01, **Figure 2B**), and TNF-α at 4 hrs (*p*<0.001,**Figure 2C**), and were inhibited by 15dPGJ2 (*p*<0.05 and *p*<0.001 respectively).

#### 3.1.3 15dPGJ2 inhibits IL-1β-induced contraction associated genes and proteins in myometrial cells but does not affect contractility

**Figure 3:**
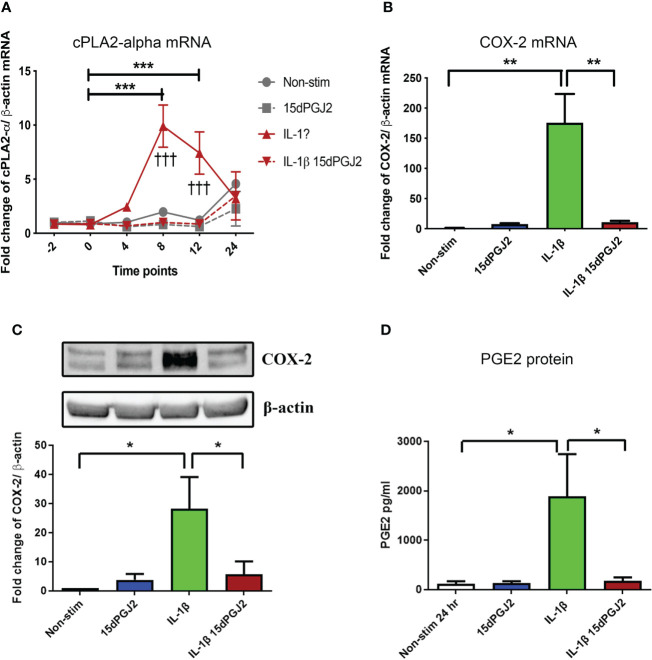
15dPGJ2 inhibits IL-1β-induced contraction associated genes and proteins in myometrial cells. Myometrial cells were pre-incubated for 2 hours with 32µM 15dPGJ2 or vehicle before stimulation with 1ng/ml of IL-1β. For cPLA2-α mRNA, cells were harvested at -2, 0, 4, 8, 12 and 24 post 1ng/ml of IL-1β stimulation. COX-2 mRNA and protein were harvested after 4 hours. mRNA level was quantified using RT-qPCR and whole cells lysates were prepared and assessed by RT-qPCR and western immunoblotting used β-actin as the loading controland reference gene. PGE2 was quantified using ELISA after 24 hours stimulation of 1ng/ml IL-1β. IL-1β-induced cPLA2-α mRNA at 8 and 12 hours and was significantly inhibited by 15dPGJ2 (p < 0.001) **(A)**. IL-1β stimulation significantly increased COX-2 production (p < 0.01) and pre-incubation with 15dPGJ2 significantly decreased mRNA (p < 0.01) **(B)** and protein expression p < 0.05). **(C)**. IL-1β stimulation increased PGE2 production and pre-incubating the myocytes with 15dPGJ2 significantly inhibited the production (p < 0.05) **(D)**. n = 3. *p < 0.05, **p < 0.01, ***p < 0.001, ^†††^p < 0.001. Data are presented as mean ± SEM.

15dPGJ2 significantly inhibited IL-1β-induced cPLA2-α mRNA expression at 8 and 12 hrs (*p*<0.001) (**Figure 3A**). IL-1β-induced COX-2 mRNA and protein expression at 4 hrs (*p*<0.01 and *p*<0.05), which was inhibited by 15dPGJ2 (*p*<0.01 and *p*<0.05 respectively, **Figures 3B, C**). Increased PGE2 production following stimulation of myocytes with IL-1β (*p*<0.05). was also inhibited by 15dPGJ2 (*p*<0.05, **Figure 3D**).

**Figure 4:**
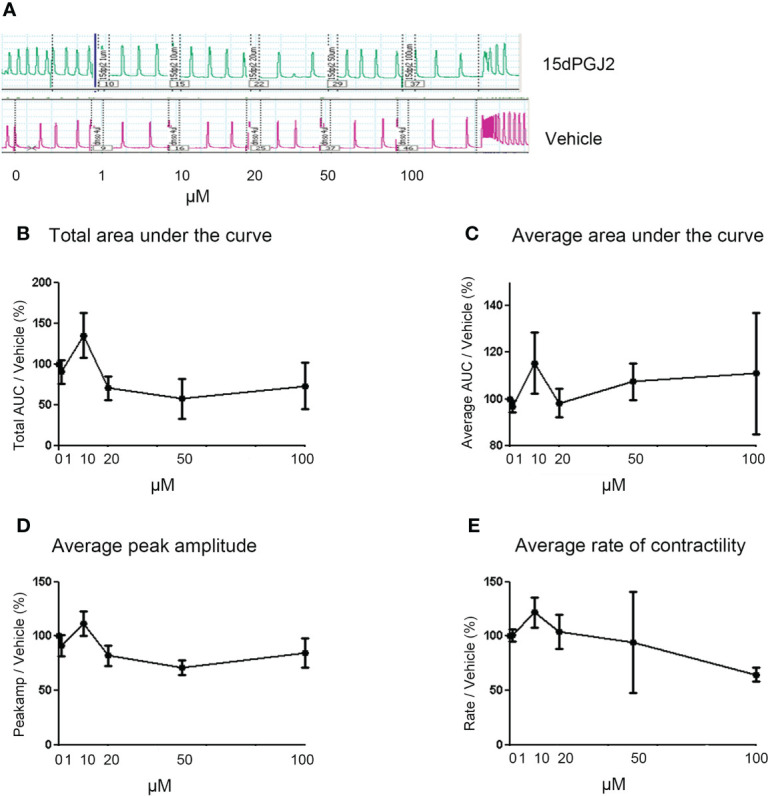
15dPGJ2 has no effect on myometrial contractility. Myometrial tissues biopsies were dissected and mounted on the myograph machine and stretched to 4g tension to achieve spontaneous contractions. After spontaneous contractions were established, the tissues were treated with 1, 10, 20, 50 and 100µM of 15dPGJ2 or vehicle control. A representative trace is shown for two 15dPGJ2 treated strips and a vehicle control strip **(A)**. A cumulative dose response with 15dPGJ2 or vehicle was performed and the effect on total and average area under the curve **(B, C)**, peak amplitude **(D)** and rate of contractility **(E)** was determined. 15dPGJ2 had no significant effect on myometrial contractility. V = vehicle. n = 3 biological replicates, each containing at least two 15dPGJ2 treated strips per biological replicate. Data are presented as mean ± SEM.

With the inhibitory effect of 15dPGJ2 seen on the main contraction associated proteins, we next evaluated the effect of 15dPGJ2 on myometrial contractility. We did not stimulate the tissue with IL-1β as we wanted to assess the tocolytic effect to model women who present with uterine contractions in threatened preterm labour where it is not possible to determine the presence of uterine inflammation. No change in total and average area under the curve, average peak amplitude or average rate of contractility was observed with 15dPGJ2 dose-response treatment compared to the vehicle control (**Figures 4A–E**).

#### 3.1.4 15dPGJ2 is non-toxic to myometrial cells

**Figure 5:**
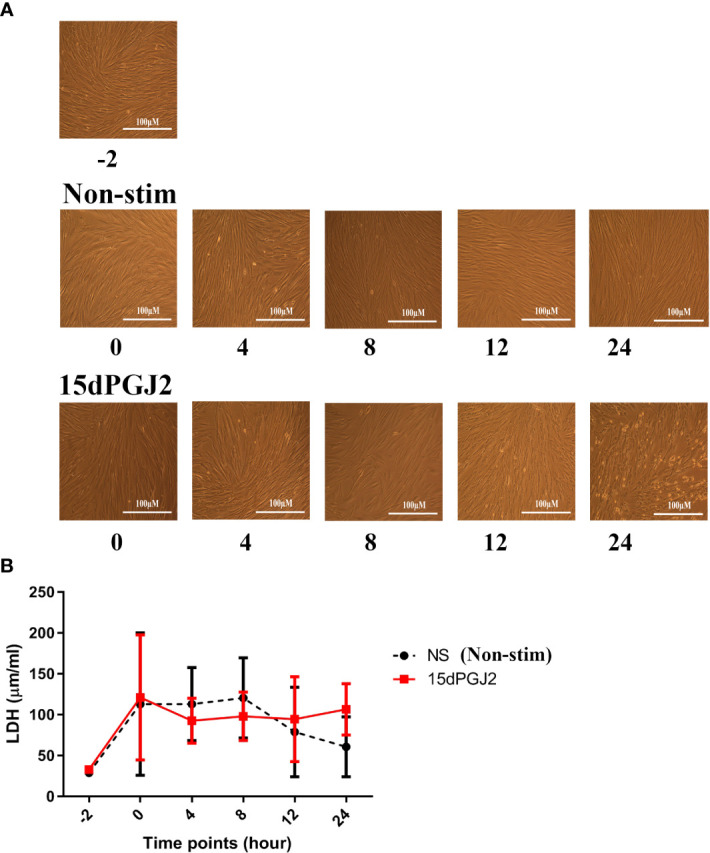
15dPGJ2 effects on myometrial cells integrity. Myometrial cells were pre-incubated with 32µM of 15dPGJ2 or vehicle for 2 hours and assessed for 24 hours. Morphology of the cells were assessed by brightfield microscopy **(A)** and LDH release into supernatant was detected at -2, 0, 4, 8, 12 and 24 hours **(B)**. No difference in LDH release was seen between timepoints or between vehicle control and 15dPGJ2 treated cells. n = 3. Data are presented as mean ± SEM. NS, Non-stim.

To confirm that 15dPGJ2 did not have any cytotoxic effects on myocytes, the morphology of the cells was examined at four hourly intervals for 12 hrs, and again at 24 hrs at the final experimental timepoint. Cell morphology appeared unchanged under examination by bright field microscopy (**Figure 5A**). Confirmation of cell health was performed by assessing LDH release in cell culture supernatant with no significant difference in LDH release between cells treated with vehicle control or 32 µm of 15dPGJ2 (**Figure 5B**).

### 3.2 Effects of 15dPG2 on vaginal and amnion epithelial cells

#### 3.2.1 15dPGJ2 has differential effects on IL-1β-induced NF-κB and AP-1 in vaginal and amnion epithelial cells

**Figure 6:**
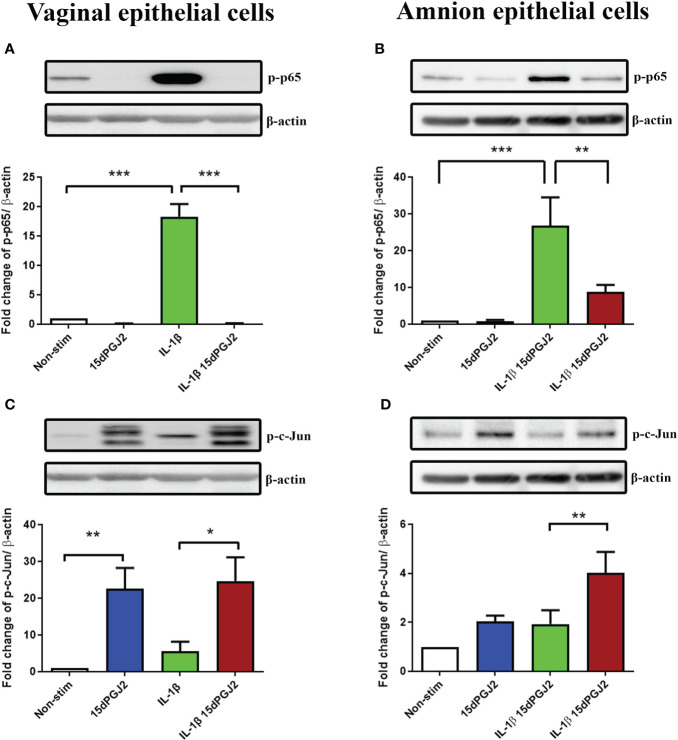
Effect of 15dPGJ2 on NF-κB and AP-1 in vaginal and amnion epithelial cells. Vaginal (VEC) and amnion epithelial cells (AECs) were pre-incubated with 32μM of 15dPGJ2 for 2 hours then stimulated with 1ng/ml of IL-1β. Whole cell lysate was used to determine serine 536-phosphorylated p65 (Ser536-p-p65) as a marker of NF-κB activation and serine 73-phosphorylated c-Jun (Ser73-p-c-Jun) as a marker of AP-1 *via* western immunoblotting. A representative blot was shown above each graph with β-actin as a loading control. Pre-incubation with 15dPGJ2 significantly inhibited IL-1β stimulated NF-κB in VECs (*p* < 0.001) **(A)** and AECs (*p* < 0.01) **(B)**. AP-1 however significantly increased after incubation with 15dPGJ2 alone as well as with 15dPGJ2 and IL-1β in VECs (*p* < 0.01) **(C)** and also in 15dPGJ2 and IL-1β treated AECs (*p*<0.01) **(D)**. *p < 0.05, **p < 0.01, ***p < 0.001. n = 3-6. Data are presented as mean ± SEM.

The promising anti-inflammatory effects of 15dPGJ2 led us to investigate this effect on VECs and AECs in an *in vitro* model of ascending inflammation. We have previously demonstrated the inhibitory effect of 15dPGJ2 on IL-1β-stimulated NF-κB in AECs. However, the effects of 15dPGJ2 on VECs has not previously been explored. IL-1β induced NF-κB activation in VECs (*p*<0.001) and AECs (*p*<0.001) and was significantly inhibited by 15dPGJ2 in both cell types (*p*<0.001 and *p*<0.01 respectively) (**Figures 6A, B**). In contrast, IL-1β did not lead to a significant increase in p-c-Jun in either cell type. However, 15dPGJ2 alone and combined with IL-1β significantly increased p-c-Jun in VECs (*p*<0.01 and *p*<0.05 respectively,**Figure 6C**). In AECs, only the combined treatment of IL-1β and 15dPGJ2 increased p-c-Jun (*p*<0.01, **Figure 6D**).

#### 3.2.2 15dPGJ2 has differential effects on inflammatory cytokine production in vaginal and amnion epithelial cells

**Figure 7:**
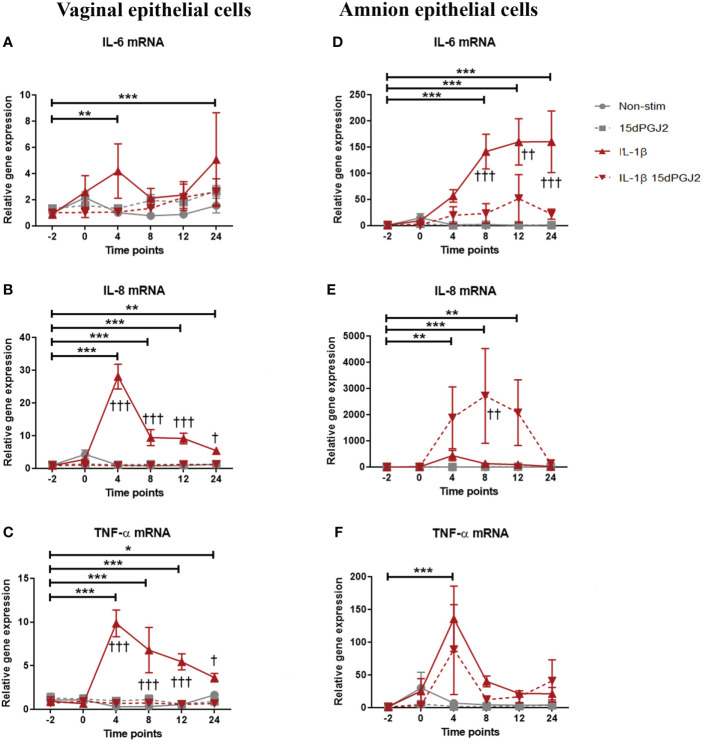
Effect of 15dPGJ2 on IL-6, IL-8 and TNF-α mRNA in vaginal and amnion epithelial cells. VEC and AECs were pre-incubated with 32μM of 15dPGJ2 for 2 hours then stimulated with 1ng/ml of IL-1β. Cells were harvested, and total RNA was quantified after -2, 0, 4, 8, 12 and 24 hours. Quantitative PCR was performed looking at IL-6, IL-8 and TNF-α mRNA level. Fold change took into account the reference gene β actin IL-1β stimulation increased IL-6 mRNA expression in VEC at 4 (**) and 24 (***) hours but no statistically significant inhibition was achieved with 15dPGJ2 pre-incubation **(A)**. IL-8 mRNA expression increased with IL-1β stimulation and this effect was inhibited by 15dPGJ2 at 4, 8, 12 (***, †††) and 24 (**, †) hours **(B)**. TNF-α mRNA also increased with IL-1β stimulation and this effect was inhibited by 15dPGJ2 at 4, 8, 12 (***, †††) and 24 (*, †) hours **(C)**. In amniocytes, IL-1β stimulation significantly increased IL-6 expression in which this effect was inhibited at 8 (***, †††), 12 (***, ††) and 24 (***, †††) hours **(D)**. Pre-incubation of 15dPGJ2 before IL-1β stimulation increased IL-8 mRNA compared to IL-1β alone (††) at 8 hours **(E)**. TNF-α mRNA expression increased at 4 hours with IL-1β stimulation (***) but the inhibition by 15dPGJ2 did not reach statistical significance **(F)**. n = 3. *p < 0.05, **p < 0.01, ***p < 0.001 = effect of IL-1β compared to -2 non-stimulated time point. ^†^ *p* < 0.05, ^††^ *p* < 0.01, ^†††^ *p* < 0.001 = effect of IL-1β 15dPGJ2 compared to IL-1β treatment alone. Data are presented as mean ± SEM.

Due to the observed differential effect of 15dPGJ2 on AP-1 activation compared to NF-κB activation in VECs and AECs, we next examined its effect on IL-1β induced pro-inflammatory cytokine production. Similar to myocytes, an increase in IL-6 (*p*<0.001), IL-8 (*p*<0.001) and TNF-α (*p*<0.001) was seen with IL-1β stimulation. Pre-treatment of VECs with 15dPGJ2 inhibited IL-1β induced IL-8 and TNF-α (*p*<0.001, **Figures 7A–C**). In contrast, IL-1β induced IL-6 (*p*<0.001), and TNF-α (*p*<0.001) production in AECs (**Figures 7D, F**), but only IL-6 was significantly inhibited by 15dPGJ2 (*p*<0.001, **Figure 7D**). Furthermore, combined treatment with IL-1β and 15dPGJ2 led to a significant and substantial increase in production of IL-8 in AECs (*p*<0.01, **Figure 7E**).

#### 3.2.3 15dPGJ2 inhibits IL-1β-induced COX-2 in amnion and vaginal epithelial cells but does not inhibit PGE2

**Figure 8:**
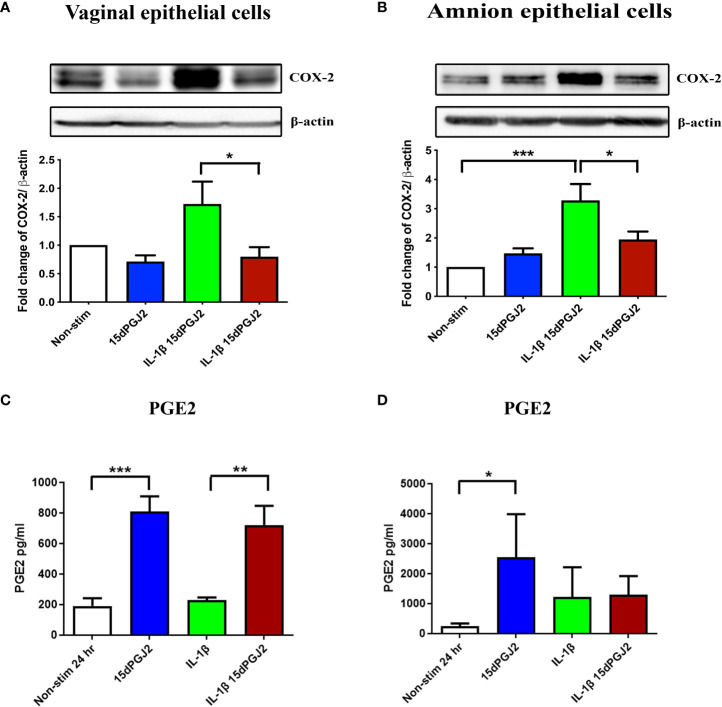
Effect of 15dPGJ2 on COX-2 and PGE2 in vaginal and amnion epithelial cells. VECs and AECs were pre-incubated with 32μM of 15dPGJ2 for 2 hours then stimulated with 1ng/ml of IL-1β for 4 hours to examine COX-2 protein expression and 24 hours to examine PGE2 production. Whole cell lysate was used to determine COX-2 protein expression and an ELISA was used to determine PGE2 concentration in cell culture supernatant. 15dPGJ2 pre-incubation significantly inhibited IL-1β stimulated COX-2 protein expression in VECs (*p* < 0.05) **(A)** and AECs **(B)** (*p* < 0.05) at 4 hours. PGE2 concentration was increased in VEC culture supernatant when treated with 15dPGJ2 (*p* < 0.001) and 15dPGJ2 plus IL-1β (*p* < 0.01) **(C)** and in supernatant of 15dPGJ2 treated AECs (*p* < 0.05) **(D)**. n=3-7. *p < 0.05, **p < 0.01, ***p < 0.001. Data are presented as mean ± SEM.

Next, we determined if there were differential effects of IL-1β and 15dPGJ2 on the expression of COX-2 and PGE2 in VECs and AECs. IL-1β induced COX-2 expression in both VECs (non-significant) and AECs (*p*<0.001), which was significantly inhibited by 15dPGJ2 pre-incubation in both VECs (*p*<0.05) (**Figure 8A**) and AECs (**Figure 8B**), (*p*<0.05). IL-1β stimulation had no significant effect on PGE2 production in either cell types. However, incubation with 15dPGJ2 alone significantly increased the production of PGE2 in VECs cells (*p*<0.001) and AECs (*p*<0.05) at 24 hrs (**Figures 8C, D**).

#### 3.2.4 15dPGJ2 increased p-JNK and p-p38, but not p-ERK

**Figure 9:**
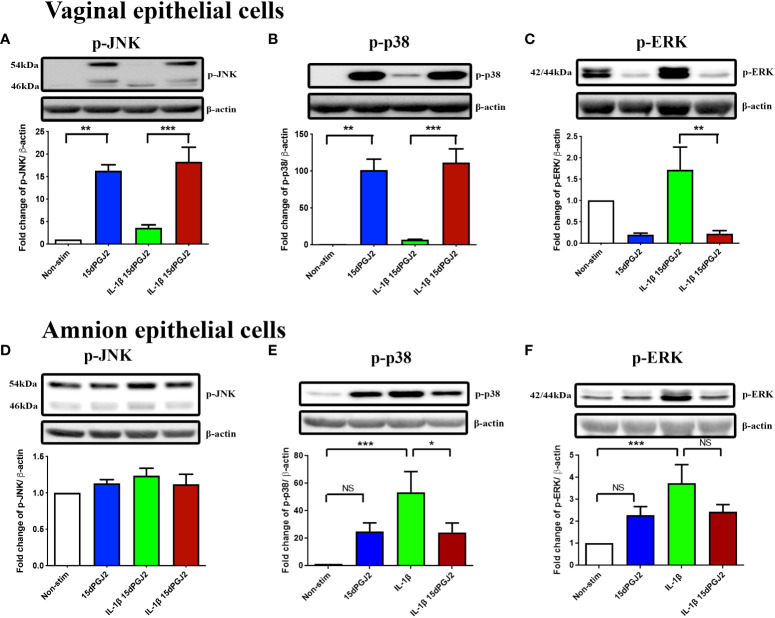
Effect of 15dPGJ2 MAPKs in vaginal and amnion epithelial cells. VECs and AECs were pre-incubated with 32μM of 15dPGJ2 for 2 hours then stimulated with 1ng/ml of IL-1β for 15 minutes. Whole cell lysate was used to determine, phosphorylated-JNK1/JNK2(Thr183/Tyr185), phosphorylated-p38(Thr180/Tyr182) and phosphorylated-ERK (Thr202/Tyr204) *via* Western immunoblotting. A representative blot was shown above each graph with β-actin as a loading control. In VECs, incubation with 15dPGJ2 alone for 2 hours significantly increased the basal level p-JNK (*p* < 0.01) **(A)** and p-p38 (*p* < 0.01) **(B)** but had no effect on p-ERK **(C)**. Treatment with 1ng/ml of IL-1β increased p-ERK, and this was attenuated by pre-incubation with 15dPGJ2 (*p* < 0.01) in VECs **(C)**. In AECs, no effect was seen on p-JNK with IL-1β and/or 15dPGJ2 **(D)**. IL-1β stimulation of AECs led to an increase in p-p-38 (*p* < 0.001) **(E)** and p-ERK (*p* < 0.001) **(F)**, the response of which were attenuated upon pre-incubation with 15dPGJ2. *p < 0.05, **p < 0.01, ***p < 0.001, NS non significant. n = 3-6.​ Data are presented as mean ± SEM.

We next examined upstream MAP kinase activation since regulation of AP-1 by MAPK signaling pathways involve p-JNK, p-p38 and p-ERK. In VECs, 15dPGJ2 treatment significantly increased p-JNK (**Figure 9A**) and p-p38 (**Figure 9B**) (*p*<0.01) levels while no effect was seen with IL-1β stimulation alone. In contrast, IL-1β-induced p-ERK was significantly attenuated by 15dPGJ2 (*p*<0.01, **Figure 9C**). Neither 15dPGJ2 nor IL-1β altered p-JNK levels in AECs (**Figure 9D**). There was a trend increase in p-p38 and p-ERK levels observed following treatment of AECs with 15dPGJ2 alone, although did not reach statistical significance (**Figures 9E, F**). However, 15dPGJ2 was shown to attenuate IL-1β induced phosphorylation of p-38 (*p*<0.05) but not ERK.

#### 3.2.5 15dPGJ2 reduced cell viability in vaginal and amnion epithelial cells ​

**Figure 10:**
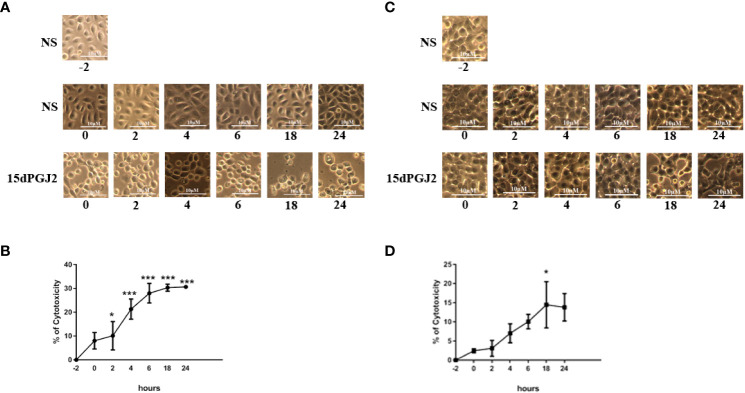
Effect of 15dPGJ2 on cell viability in vaginal and amnion epithelial cells. VECs and AECs were incubated with or without 32μM of 15dPGJ2 and the morphology of the cells were assessed by brightfield light microscopy at -2, 0, 2, 4, 6, 18 and 24 hours. 15dPGJ2 or vehicle control was inserted in the corresponding wells at -2 timepoint following image and supernatant capture. The culture medium at each time point was used to detect cell viability using the MTT assay. The morphology of VECs changed from a healthy polygonal shape to an abnormal round shape compared to the control **(A)**. The effect of 15dPGJ2 on cell viability was detected as early as 2 hours (*p* < 0.05), and a steady change was seen at 4, 6, 18 and 24 hours (*p* < 0.001) **(B)**. In AECs, the morphological shape of the cells started to change from an ovoid, slab stone- and cobblestone-like pattern to a stretched star-like pattern at 18 hours compared to the control **(C)**. The cell viability of amnion epithelial cells was significantly changed by 18 hours in 15dPGJ2 treated cells (*p* < 0.05) **(D)**. *p < 0.05, ***p < 0.001, NS non significant. n = 3.​ Data are presented as mean ± SEM.

Examination of the morphological appearance of VECs and AECs under light microscopy during the experimental time points following administration of either vehicle control or 15dPGJ2,indicating changes suggestive of 15dPGJ2 induced reduction in cell integrity (**Figures 10A**, **C** respectively). MTT assaying confirmed a reduction in cell viability from the 2 hr time point in VECs (*p*<0.05, **Figure 10B**), and in AECs which reached statistical significance at 18 hrs post treatment (*p*<0.05, **Figure 10D**).

## 4 Discussion

Parturition is considered to be an inflammatory process, with term labour being a physiological process. In contrast, preterm labour is a pathological process whereby premature activation of biochemical and inflammatory pathways occur (4, 41). The presence of infection and or inflammation is seen in 25-40% of all preterm births (6), and in up to 80% of extreme preterm births (42). We and others have shown that the presence of certain vaginal microbial communities increase the risk of preterm birth (43), which is likely to be due to the combined effect of local inflammation and ascending infection/inflammation (44, 45). Furthermore, inflammation in the absence of infection, known as ‘sterile inflammation’, has also been widely associated with PTB (46, 47). Activation of the transcription factors NF-κB and AP-1 initiate a proinflammatory cascade of cytokines, chemokines, COX-2, prostaglandins, and matrix metalloproteinases (14, 48, 49) which participate in several processes of labour including cervical remodelling, uterine contractility, and rupture of fetal membranes (50). Our study reports on the effects of 15dPGJ2 on NF-κB, AP-1, cytokines, COX-2, and prostaglandins. Whilst we demonstrate inhibitory effects in myocytes, we demonstrate unfavourable stimulatory effects on pro-inflammatory and pro-labour mediators and reduced cell viability in VEC and AECs.

One of the challenges of developing new therapeutic strategies for pregnancy related conditions is the desire to deliver drugs in a way that avoids transport to the fetus, and to target relevant gestational tissues, such as the myometrium or the cervix. Nanomedicine based technology for drug delivery has been used to deliver histone deacetylase (HDAC) inhibitors *via* vaginal pessary in the mouse and leads to a reduction in PTB rates (51). Several groups have purified and encapsulated 15dPGJ2 into nanoparticles and both *in vitro* and *in vivo* experiments have confirmed anti-inflammatory effects (52–54). For example, 15dPGJ2 loaded nanoparticles inhibit surgically induced inflammation in rat femurs as demonstrated by a reduction in tissue IL-6, IL-1β, and TNF-α mRNA (54). Intraperitoneal delivery of 15dPGJ2 solid lipid nanoparticles leads to a reduction in neutrophil migration and reduced IL-1β concentrations in peritoneal fluid of LPS treated mice (53). The lack of tocolytic effect seen in myometrial strips in our study would not support the development of targeted drug delivery of 15dPGJ2 to the uterus in humans.

We have previously shown that intra-amniotic co-administration of LPS with 15dPGJ2 delays PTB and increases pup survival in the mouse (20). This was demonstrated to involve inhibition of myometrial p-p65, p-IκBβ, and JNK activity, a reduction in p-cPLA2, COX-2 expression, and a reduction in the proinflammatory chemokines CCL2 and CXCL1 when mice were co-treated with 15dPGJ2 compared to LPS alone. Consistent with this murine model, we have demonstrated with the current study that 15dPGJ2 is also able to inhibit IL-1β induced pro-contractile and pro-inflammatory and mediators in human cultured primary myocytes. However, we acknowledge that some of our results have large error bars and should be interpreted with his in mind. Despite this, our results are consistent with our previous findings that 15dPGJ2 inhibits IL-1β and PMA-induced p-p65 in myocytes, AECs and PBMCs (26, 39). In addition, we demonstrated inhibition of IL-1β induced AP-1 activation in myocytes. AP-1 proteins (cFos, FosB, c-Jun, JunB and JunD) are expressed in human myometrium (31), and term labour is associated with an increase in cFos, and JunB expression (33). *In vitro* assays show that AP-1 transcriptional activity is increased with exposure to TNF- α (33). Both NF-κB and AP-1 have been shown to directly regulate IL-1β- induced IL-8 gene expression in myocytes cultured from human myometrium at term (15). Furthermore, we have demonstrated that IL-1β-induced mRNA expression of IL-6, TNF-α, and IL-8 occurs in association with increased p-p65 and p-c-Jun protein expression. It is therefore unsurprising that 15dPGJ was able to inhibit IL-1β- induced cytokine production.

Despite inhibition of IL-1β induced cPLA2, COX 2 and PGE2 in myocytes, the lack of inhibitory effect on myometrial contractility may have been due to the limitation of the *ex vivo* contractility experimental model. Myometrial strips do not contract for longer than 8 hrs in our model, and therefore it is not possible to determine the longer term effect of 15dPGJ2-mediation of PGE2 production and contractility. Another possible limitation was that we did not test the effect of 15dPGJ2 on IL-1β treated myometrial strips. Our *ex – vivo* contractility model was designed to determine if 15dPGJ2 had tocolytic effects once contractions had commenced and in the absence of inflammation, since the majority of women who present in threatened preterm labour are already contracting, and it is not always possible to establish if inflammation is present before commencing tocolytic therapy.

In preterm birth, the presence of intra-amniotic infection and inflammation is most commonly due to ascending from the vagina (22). Therefore, when exploring new therapeutic targets, it is also essential to explore effects on both VECs and AECs. We acknowledge that AECs were cultured from tissue taken from women at term rather than preterm gestations, and that a cell line was used to study the effects of 15dPG2 on VECs. This may limit the application of our results to the use of 15dPGJ2 in preterm women. Our justification for using pre labour healthy term samples (and a cell line for VECs), was to limit variation in cellular responses. As observed in myocytes, 15dPGJ2 was able to inhibit IL-1β-induced NF-κB activation in both VECs and AECs. However, a differential response in AP-1 activation was seen in the epithelial cells. Despite 15dPGJ2 inhibiting p-c-Jun in myocytes, it led to increased phosphorylation of c-Jun in both epithelial cell types, but more significantly in VECs. Despite many studies demonstrating 15dPGJ2 driven inhibition of AP-1 activation (55–58), there are also several studies confirming 15dPGJ2 driven activation, predominantly in epithelial cells. AP-1 activation, as determined by increased AP-1 DNA binding activity, is increased in both epithelial colonic and breast cancer cell lines following treatment with 10 and 30µm of 15dPGJ2 respectively (59, 60).

The mechanism for 15dPGJ2 induced activation of AP-1 is unclear. AP-1 regulation by the mitogen-activated protein kinase (MAP) signal transduction pathways occurs *via:* extracellular signal-regulated kinases (ERKs), c-Jun N terminal kinases (JNKs), and p-38 mitogen activated protein kinases (p38). Studies have demonstrated variable effects of 15dPGJ2 on the MAP kinases, but most report on activation rather than inhibition. 15dPGJ2 can induce JNK activation (61), p-p38 (62) and p-ERK (63, 64) expression, but has also been reported to inhibit JNK (65), p-38 (66) and p-ERK (67) expression. The differential effect is likely to be dependent on multiple variables including dosing time course, concentration, and cell type. In our study we saw a trend in activation of p-p38 and p-ERK in AECs treated with 15dPGJ2 alone, yet 15dPGJ2 was able to inhibit the stimulatory effects of IL-1β. In contrast, we observed a significant and substantial increase in p-JNK and p-p38 expression in VECs following pre-treatment with 15dPGJ2. Without the use of specific inhibitors and/or knockdown experiments, our results are purely observational, rather than mechanistic and therefore, we cannot conclude how AP-1 is being regulated in response to 15dPGJ2 in our study. 

We showed that 15dPGJ2 inhibited IL-1β induced cytokine production in VECs and AECs. Although we acknowledge that the cytokine responses were subject to large error bars in some cases, which may limit the validity of some data. Lappas et al. examined the effects of 30 µm 15dPGJ2 and the synthetic PPAR-γ troglitazone on lipopolysaccharide induced cytokine production in placental, amnion and choriodecidual explants (68). Cytokine production was reduced in all tissue types with both agents, yet only 15dPGJ2 led to attenuation of NF-κB DNA binding activity. Berry et al. showed differential and dose dependant effects of 15dPGJ2 and the PPAR-γ ligand rosiglitazone on IL-1β induced pro-inflammatory and pro-labour mediator production in amnion derived WISH epithelial cells (69). These studies suggest that the downstream effects of 15dPGJ2 differ depending on which pathway is targeted and is likely to be influenced by cell type and the concentration of 15dPGJ2.

The enzyme COX-2 is the rate limiting step for prostaglandin production and plays a key role in fetal membrane rupture and cervical remodelling in the process of labour. Clinically, PGE2 is used as a vaginal pessary to ripen the cervix and induce labour at term, whereas the non -selective COX inhibitor Indomethacin has been shown to delay preterm labour (70). Both NF-κB and AP-1 have been shown to transcriptionally regulate COX-2 expression (71). In this study, IL-1β induced COX-2 expression was inhibited by 15dPGJ2 in all cell types at 4 hrs, yet at 24 hrs PGE2 production was substantially increased in VECs and AECs, but not myocytes, when treated with 15dPGJ2. The capacity for 15dPGJ2 to modulated COX-2 expression appears to be cell type specific. For example, in astrocytes it inhibits COX-2 expression yet has no effect on microglia COX-2 expression. In contrast, 15dPGJ2 has been reported to increase expression in osteosarcoma cells and breast cancer cells (34, 62). It is plausible that increased transcription of COX-2 in VECs and AECs occurred at a later timepoint than that assessed during this study. In support of this, 15dPGJ2 induced COX-2 expression has been reported to be first detectable at 6 hrs, increasing further at 12 hrs, with maximal expression seen at 24 hrs (72). Furthermore, it is likely that transcriptional regulation was *via* AP-1 and not NF-κB since the increase in PGE2 was seen in VECs and AECs but not in myocytes, although not conclusive in the without the use of specific inhibitors.

Despite not seeing any obvious morphological changes to myocytes treated with 15dPGj2, we saw a significant change in morphology of both amnion and vaginal epithelial cells. We also saw a significant reduction in cell viability as demonstrated by the MTT assay. Previous studies have shown similar effects on cell viability with a dose and time dependent effect (73, 74). Keelan et al. showed that 15dPGJ2 reduced cell viability, using the MTT assay, from 4 hours onwards, and with increasing the concentration up to 30 µm in amnion-like WISH cells (73). They also demonstrated morphological changes consistent with apoptosis from 2 hours, and biochemical changes like caspase activation and substrate cleavage from four hours post incubation. Since rosiglitazone had no effect on cell viability, they concluded this was likely to be a mechanism independent of PPAR-γ activation. We did not pursue exploring the role of PPARs in the 15dPGJ2-mediated effects demonstrated in this study, neither did we explore the explicit role of the MAP kinases with specific agonists/inhibitors. Whilst this may be seen as a limitation to our study, we felt that the unfavourable effects of 15dPGJ2 on cell viability rendered 15dPDJ2 an unlikely therapeutic agent and therefore we did not explore additional *in vitro* molecular targets.

**Figure 11:**
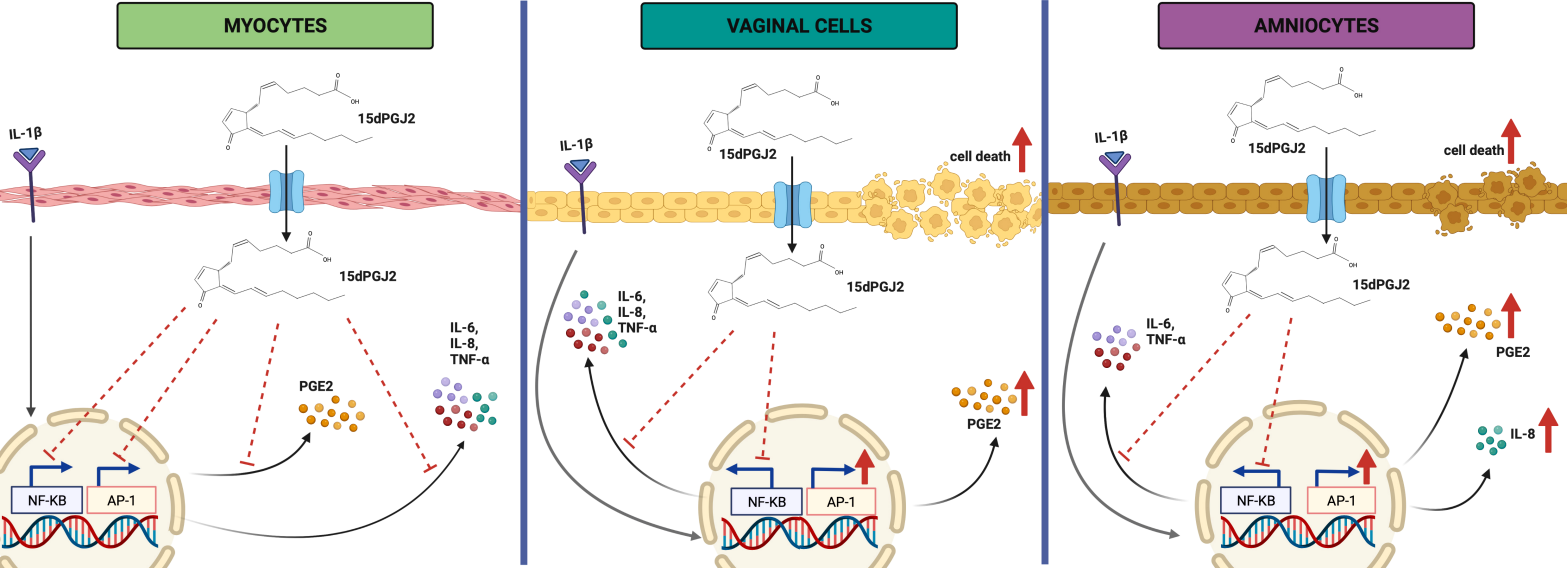
Illustration on the effect of IL-1β and 15dPGJ2 on myocytes, vaginal and amnion epithelial cells. Pre-incubation of 15dPGJ2 in myocytes for 2 hours inhibited IL-1β induced activation of the transcription factors NF-κB and AP-1. This led to inhibition of cytokine expression and PGE2 production. In VECs and AECs, 15dPGJ2 inhibited IL-1β-stimulated NF-κB but led to activation of AP-1. A downstream inhibition of the pro-labour cytokines IL-6, IL-8, and TNF-α was seen in VECs. In contrast upregulation of IL-8 was seen in AECs. PGE2 production was increased both VECs and AECs following treatment with 15dPGJ2, and this may be due to AP-1 activation. In addition, increased cell death was seen in VECs and AECs with 15dPGJ2 treatment. We conclude that 15dPGJ2 has differential effects depending on cell type, and due to its ability to increase the production of pro-labour and pro- inflammatory mediators, we would not advocate its development as a novel therapeutic strategy for preterm birth prevention. Created with BioRender.com

In summary, we have reported on the cell type specific effects of 15dPGJ2 on pro-inflammatory and pro-labour mediators in an *in vitro* model of local and ascending and intrauterine inflammation. Whilst 15dPGJ2 had promising inhibitory effects on IL-1β induced pro-inflammatory and pro-contractile mediators in myocytes as summarised in **Figure 11**, there was no inhibition of myometrial contractility, and there was activation of AP-1, reduced cell viability, and increased production of PGE2 in both VECs and AECs (**Figures 11B, C**), and additionally an increase in IL-8 in AECs. We conclude that 15dPGJ2 is therefore unlikely to be an effective therapeutic agent in humans for preventing inflammation and/or infection induced preterm labour.

## Data availability statement

The raw data supporting the conclusions of this article will be made available by the authors, without undue reservation.

## Ethics statement

The studies involving human participants were reviewed and approved by Riverside Research Ethics Committee (Ref 3358) and Hammersmith, Queen Charlotte’s & Chelsea Hospitals Research Ethics Committee (Ref 2002/628). The patients/participants provided their written informed consent to participate in this study.

## Author contributions

LS, ZR, TT, DM, and PB were responsible for the conception and design of the study. ZR, SK, YL, and LS conducted experiments and data analysis. All figures and tables were created by ZR and LS. ZR drafted the manuscript and all authors critically reviewed it. All authors contributed to the article and approved the submitted version.

## Funding

This study was funded by Majlis Amanah Rakyat (MARA) (MARA Ref: 3304082377500), Malaysian Government Agency. LS was funded by a Wellbeing of Women Research Training Fellowship (Grant Ref 148), a National Institute for Health Research (NIHR) Clinical Lectureship and Genesis Research Trust and is currently a Parasol Foundation Clinical Senior Lecturer in Obstetrics. DAM was supported by the Medical Research Council (Grant Ref MR/L009226/1). This work was also supported by the National Institute for Health Research Comprehensive Biomedical Research Centre at Imperial College Healthcare NHS Trust and Imperial College London (Grant Ref P45272). This publication presents independent research funded by the National Institute for Health Research (NIHR). The views expressed are those of the authors and not necessarily those of Imperial College, the NHS, the NIHR or the Department of Health.

## Acknowledgments

We would like to thank the study participants from Imperial College Healthcare NHS Trust for their contribution. We would also like to thank Xintong Li for her contributions as part of her BSc project at Imperial College London.

## Conflict of interest

The authors declare that the research was conducted in the absence of any commercial or financial relationships that could be construed as a potential conflict of interest.

## Publisher’s note

All claims expressed in this article are solely those of the authors and do not necessarily represent those of their affiliated organizations, or those of the publisher, the editors and the reviewers. Any product that may be evaluated in this article, or claim that may be made by its manufacturer, is not guaranteed or endorsed by the publisher.

## Abbreviations

15dPGJ2, 15-Deoxy-Delta-12,14-prostaglandin J2; AP-1, activator protein-1; BPE, bovine pituitary extract; CaCL_2,_ calcium chloride; COX-2, cyclooxygenase-2; cPLA2-α, cytosolic phospholipase A2- alpha; Cx43, connexin 43; DMT, Danish Myo Technology AS; DMEM, dulbecco’s modified eagle’s medium; EGF, epidermal growth factor; FCS, fetal calf serum; IL, interleukin; IL-1β, interleukin-1β; KSFM, keratinocytes serum free medium; LDH, lactate dehydrogenase; MTT, 3-(4,5-dimethylthiazol-2-yl)-2,5-diphenyl-2H-tetrazolium bromide; NF-κB, nuclear factor‐κB; p-JNK, phospho-c-Jun N terminal kinase; p-p38, phospho-p-38 mitogen activated protein kinase; p-ERK, phospho-extracellular signal-regulated kinase; PGE2, prostaglandin E2; TNF‐α, tumour necrosis factor‐alpha.
